# Prevalence and treatment of allergies in rural areas of Bavaria, Germany: a cross-sectional study

**DOI:** 10.1186/s40413-018-0218-z

**Published:** 2018-11-19

**Authors:** Danielle Boehmer, Barbara Schuster, Julia Krause, Ulf Darsow, Tilo Biedermann, Alexander Zink

**Affiliations:** 1 Klinik und Poliklinik für Dermatologie und Allergologie am Biederstein, Technical University of Munich, Biedersteiner Str. 29, 80802 Munich, Germany; 2 Clinical Unit Allergology, Helmholtz Zentrum München, German Research Center for Environmental Health GmbH, Neuherberg, Germany

**Keywords:** Allergies, Bavaria, Prevalence, Treatment, Allergy, Rural area

## Abstract

**Background:**

There is a high prevalence of allergies in Germany, with approximately 20% of the population having at least one allergy and only about 10% of these being treated adequately. We conducted a cross-sectional study in a rural area of Bavaria (Southern Germany) to evaluate the prevalence of allergies and their treatment, because research regarding this topic is currently limited.

**Methods:**

Data were collected in 10 offices of non-dermatological doctors using a self-filled questionnaire to ask participants about allergies and treatment.

**Results:**

A total of 641 questionnaires were appropriate for analysis. The prevalence of allergies in the Bavarian countryside was higher than that reported for Germany (37.3% vs. 20.0%). Furthermore, almost a third (30.4%) of allergies were not treated at all. The most frequently consulted therapist was found to be a general practitioner.

**Conclusions:**

Based on the study results, there is a need for prevention programs and establishment of treatments for certain allergies to minimize long-term health effects. Moreover, more studies are needed to analyze the prevalence of allergies in farmers who had a higher prevalence of allergies compared to previously reported prevalence in literature reviews.

**Trial registrations:**

The study was approved by the ethical review committee of the Technical University Munich (*EC number 548/16S).*

## Background

Allergies are a common health problem worldwide [1]. Exact figures on the prevalence are not available, estimations vary between 20 and 40% [2]. Not only do they influence the quality of life of affected patients, they also represent a significant financial burden to the health systems [3].

According to the position paper of the Environmental Medicine Commission of the Robert Koch Institute (RKI), a German federal government agency and research institute responsible for disease control and prevention, the life time prevalence of allergic diseases in Germany in adults was 8.6% for asthma, 14.8% for hay fever, 3.5% for atopic dermatitis, 8.1% for contact dermatitis, 4.7% for food allergies, and 2.8% for insect venom allergies [3]. According to the RKI, approximately 20% of all German adults and 26% of all children and adolescents are affected by at least one allergy, but only 10% of the allergies are treated appropriately [4]. According to data collected by the European Centre for Allergy Research Foundation, the annual average costs for a patient with hay fever are as high as 1545€. Furthermore, the foundation emphasizes the necessity of correct treatment of rhinitis without which the risk for developing bronchial asthma will increase [4]. The costs for patients who suffer from asthma and rhinitis is six-fold and as high as the treatment for only rhinitis. If allergies were treated correctly in Europe, approximately 84 million € could be saved per year [4]. Exact data regarding the prevalence of allergies and allergic diseases are difficult to collect because the definition of allergy is variable and broad-based epidemiological studies are hard to conduct. Furthermore, studies to compare the country-side and urban areas are rare [5, 6].

In the past, various researchers showed that farming and growing up on a farm in rural areas reduces the risk of having an allergy [5, 7–10]. Figures for rural areas in Germany had not been published to date. A small portion (2.3%) of our study population were farmers; therefore, we also assessed their allergies in view of this hypothesis.

There have been various theories to explain why allergies are increasing worldwide, especially amongst the farming population. One of the theories is the hygiene hypothesis, which states that exposure to microbials at a young age can lead to a lower risk of developing an allergy than without exposure. Due to our modern sanitized living conditions, our contact with microbials has greatly decreased. This leads to the theory that the change in the microbiome due to lifestyle might be a reason for the increasing prevalence of allergies. The understanding of the microbiome and the development of treatments to influence this could significantly change the prevalence of allergies in the future [11, 12].

The aim of this study was to evaluate the prevalence of allergies in rural Bavaria and compare these numbers to data collected across Germany [13]. Furthermore, we evaluated the treatment of allergies in rural areas.

## Methods

This study was a cross-sectional study conducted using a paper-based self-filled questionnaire in the Bavarian Forest as part of the previously published WALD (the official name of the study conducted in the past, it means forrest in German as it was conducted in an area of Bavaria which has lots of forrests) study ([4, 14]. The data were collected during the first quarter of 2017. The anonymous questionnaires were distributed in 10 offices of non-dermatological doctors in rural Bavaria, Southern Germany. Locations of the doctors’ offices were in the Bavarian forest (rural districts: Cham, Freyung-Grafenau, Passau, and Regen), an area officially declared as countryside by the federal government of Germany [14, 15]. The doctors were general practitioners (*n* = 1) and specialists in internal medicine (*n* = 2) and orthopedics and surgery (*n* = 7). They were chosen from throughout the region to ensure adequate representation. To minimize selection bias, both patients and their company were eligible for participation in this study. The minimum age for participation was 18 years. Patients had to give written consent. The study was approved by the ethical review committee of the Technical University Munich (*ethical committee (EC) number 548/16S*).

In the questionnaire, the participants were asked if they suffered from an allergy, whether it was treated, and what treatment was administered. The examined allergies were pollen, animal hair, bee venom, wasp venom, contact, food, drug, house dust mite, and other allergies. Participants were asked to indicate whether they self-treated their allergy or if their allergy was treated by a general practitioner (GP), a dermatologist, an ear, nose, and throat specialist (ENT), an alternative practitioner, or not treated at all. Furthermore, the questionnaires were used to determine the age and sex of the participants and their current or former profession. This additional information allowed to compare the prevalence of allergies based on various jobs (e.g., indoor vs. outdoor jobs. Participants were grouped into 4 age groups (18–29 years, 30–44 years, 45–64 years, and ≥ 65 years), following the example of the “Gesundheit in Deutschland aktuell study” conducted by the RKI [4, 14].

Assistance to fill in the questionnaire was provided by the medical practitioners, if necessary. Filled anonymous questionnaires were sent back for digitalization with the program EpiInfo (statistical software for epidemiology developed by the Centers for Disease Control and Prevention, Atlanta, Georgia, USA). Some questionnaires were randomly chosen to monitor the accuracy of digitalization.

The data were analyzed using descriptive statistics. Odds ratios (ORs) were calculated to compare the risk of allergy between men and women and different age and professional groups. To determine significance between relationships, 95% confidence intervals of the ORs were determined. The data were analyzed using IBM SPSS version 24 (IBM cooperation, Armonk, USA).

## Results

From January to March 2017, a total of 641 patients (59.7% women, 40.3% male; mean age and standard deviation, 50.5 ± 15.1 years; range, 18–86 years) participated. Of these, 67.2% had indoor jobs (e.g., office jobs; *n* = 387), 24, 7% were pensioners (*n* = 154), 14.4% were housewives/house husbands (*n* = 83), 7.3% were construction workers (*n* = 42), 2.3% were farmers (*n* = 13), 2.4% were students or scholars (*n* = 14), 1.9% were unemployed (*n* = 11), and 5.9% had other jobs (*n* = 38; Table 1).

**Table 1:** Study population

Number of participants	641
Gender	
Men	40.3% (*n* = 258)
Women	59.7% (*n* = 383)
Missing information on gender	> 0.1%(*n* = 10)
Age	
Mean	50.5 years
Standard deviation	15.1 years
Range	18–86 years
18–29 years	11.4% (*n* = 73)
30–44 years	17.3% (*n* = 111)
45–64 years	53.7% (*n* = 344)
> 64 years	17.6% (*n* = 113)
Missing information on age	> 0.1% (*n* = 3)
Profession^a^	
Indoor workers (office etc.)	60.4% (*n* = 387)
House wives / house husbands	12.9% (*n* = 83)
Construction workers	6.6% (*n* = 42)
Students and scholars	2.2% (*n* = 14)
Farmers	2% (*n* = 13)
No employment	1.7% (*n* = 11)
Hunters	1.1% (*n* = 7)
Others	5.3%(*n* = 34)
Missing information on profession	10.9% (*n* = 70)
Retirement	
Pensioners	24% (*n* = 154)
Missing information on retirement	> 0.1% (*n* = 22)

^a^18 participants indicated having two professions, 1 reported having three professions

The response rate for this current sample is unknown. The data for this study were collected within the scope of the WALD-study (response rate, 77.8%) [14]. However, only 718 of the 1007 participants of the WALD-study had actively received the questionnaire regarding allergies that was used in our current study. The number of people who had saw the questionnaire at the doctor’s offices but did not complete it was unknown. In addition, participants recruited by dermatologists (*n* = 77) were excluded from this analysis to minimize selection bias.

### Prevalence of allergies

**Fig. 1:**
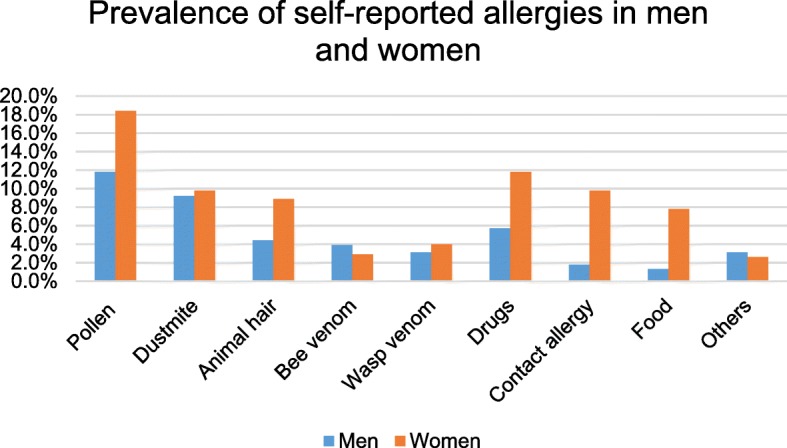
Prevalence of self-reported allergies stratified by gender

Approximately 37.3% of the whole cohort had at least one allergy, and 28.1% and 43.2% of all questioned men and women, respectively, stated that they had allergies. Women had a significantly higher risk of allergies than men (OR, 2; confidence interval [CI], [1.4; 2.8]). Furthermore, women showed a significantly higher risk for most examined allergies, especially for food allergies (OR, 6.3; CI, [1.9; 21.1]) and contact allergies (OR, 6.1; CI, [2.1; 17.4]) compared to men (Fig. 1). However, the risk for bee venom allergy (OR, 0.7; CI, [0.3; 1.8]) and other allergies (OR, 0.8; CI, [0.3; 2.3]) was lower in women, and there was no significant difference in the risk for wasp venom allergy (OR, 1.3; CI, [0.53; 3.3]) and house dust mite allergy (OR, 1.1; CI, [0.6; 1.9]).

**Fig. 2:**
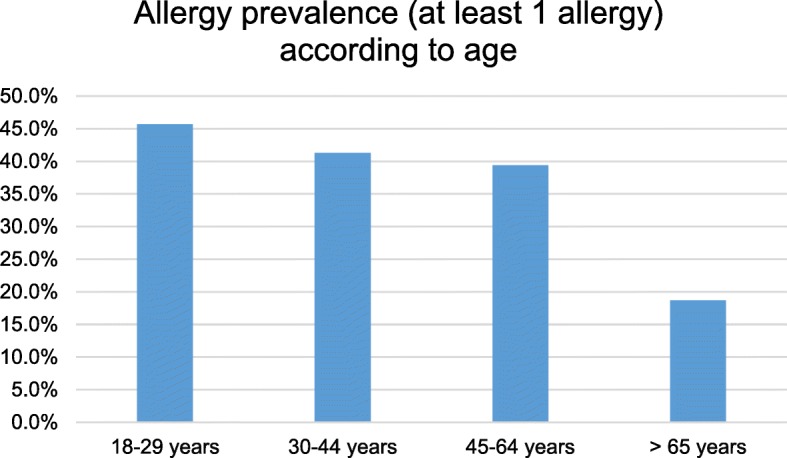
Prevalence of allergies according to age

In the study population, the highest prevalence of allergy was amongst the young population aged 18–29 years (45.7% had at least one allergy). The lowest prevalence was observed in the population aged > 65 years (18.7%; Fig. 2). The most common allergy was pollen allergy (16.0%), and the least common allergies were bee venom (3.4%) and other allergies (2.7%; Fig. 2). Pollen allergy was the most common allergy in the age groups 18–29 years (25.7%), 30–44 years (18.3%), and 45–64 years (16.1%). In contrast, the most common allergy found in participants aged ≥65 years was drug allergies (6.6%).

Indoor workers (OR, 1.5; CI, [1.0; 2.2]), farmers (OR, 1.3; CI, [0.3; 4.9]) and unemployed participants (OR, 1.3; CI, [0.4; 4.5]) were affected by allergies the most. Pensioners (OR, 0.5; CI, [0.3; 0.8)], construction workers (OR, 0.4; CI, [0.2; 0.9]), and hunters (OR, 0.3; CI, [0.0; 2.7]) were affected the least. However, only the ORs for pensioners and construction workers proved to be significantly < 1. Regarding the different allergies, the only significant finding was that pensioners had a lower risk of pollen allergy (OR, 0.4; CI, [0.2; 0.8]) compared to participants in other occupations.

### Treatment of allergies in rural Bavaria

**Fig. 3:**
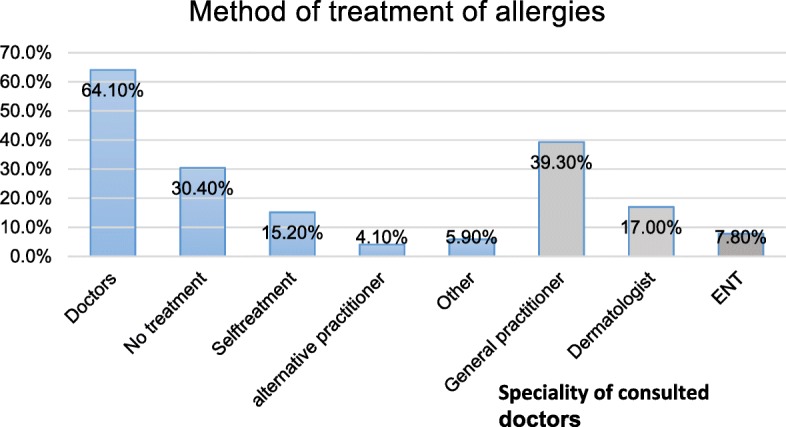
Display of consulted doctors or methods of treatment of allergies (at least one method of treatment)

**Fig. 4:**
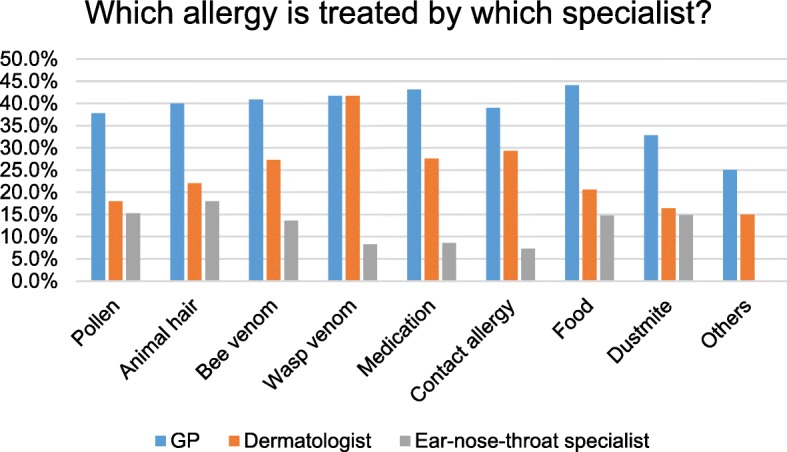
Treatment of allergies by GPs (general practitioners), dermatologists and ENTs (ear nose throat specialists)

Based on our data, 30.4% of all allergies were not treated at all. A GP was consulted most frequently to treat the allergy (39.3%), followed by a dermatologist (17.0%) and ENT doctor (7.8%), while 15.2% stated self-treatment of their allergy (Fig. 3). The most commonly treated allergy (including self-treatment) was food allergy (79.3%), and the least treated was contact allergy (62.2%). The most common allergies that were treated by a dermatologist, ENT doctor, or GP were animal hair and wasp venom allergy (both 61.9%; Fig. 4). Contact allergies (48.6%) and other allergies (43.8%) were treated the least frequently by GPs, dermatologists, and ENT doctors (Fig. 4).

## Discussion

According to the study results, allergies are common in the rural areas of Southern Germany, and a large proportion of these allergies is not appropriately treated. We found that women had a higher prevalence of allergies than men, and similar findings were also found in various studies conducted in the past [4]. A reason for this might be that women tend to consult doctors more frequently than men do. Although this association has not been directly mentioned in the literature, there are assumptions regarding this. However, inconsistent and weak findings for this hypothesis was found in a study conducted by Hunt et al. in 2011 to evaluate whether women consult doctors more frequently for headache and backpain [16].

In a study conducted in 2008–2011 to examine blood samples for specific immunoglobulin E (IgE) antibodies, men were more frequently sensitized to at least one allergen than women [4, 17]. A question that does arise is whether men tend to ignore or are less aware of their allergies than women. Furthermore, women may not necessarily have an allergy but think that they do [18]. Previous researchers have emphasized the influence of the relationship between female steroid sex hormones and the existence of allergies. The fact that the prevalence of allergies is usually higher in men before puberty and higher in females after puberty, underlines the possible relationship between female hormones and allergies. Reasons for this might be that estrogen receptors are found on numerous immunoregulatory cells [19]. It has been suggested that estrogen can act to move the immune response towards an allergic response directly or via the modulation of immunologic pathways [20]. Estrogen can influence immune cells to favor an allergic response by promoting Th2 polarization, which can lead to class switching of B cells to then produce IgE. Nevertheless, the roles of the hormones have not fully been elucidated and further studies are needed [21].

Another interesting finding was that allergies were more common in the younger population. The younger generation is often better informed than the elderly, which could explain a higher prevalence of allergies (e.g., histamine intolerance) in that group. The older generation might not know that an allergy could be an explanation for their symptoms. Furthermore, another reason could be that individuals who are aware that they have a certain allergy may avoid the allergen (e.g., food that causes allergic symptoms) during most of their life, which can result in less pronounced allergic episodes. Another approach to explain this is that the current younger generation is living in an environment that is different to that lived by the older generation many years ago. The hygiene hypothesis, which was established a few years ago, suggests that allergies develop easily when there is less contact with microbials. Dr. von Mutius found that the development of asthma and allergies was significantly lower in children who were exposed to the microbial environment compared to those who were not exposed [22, 23]. Furthermore, there are many factors that can influence allergic diseases during aging, such as genetic, epigenetic, immunosenescence, external risk factors, internal diseases, and medications. Immunosenescence is the change in immune function with aging. Immunosenescence involves modification and remodeling of tissue structure. Many functions decline with aging, while other functions can become more active. A senescent immune system can show impaired interactions between the innate and adaptive immune responses. Interaction between the different components of the immune system can determine a complicated immunological profile in the elderly population [24]. Furthermore, patients who are older often take many medications, which can also interfere with allergies and allergic reactions. Nevertheless, the literature still lacks sufficient information regarding allergies and their prevalence in the elderly population. Due to our increasing understanding of the molecular and genetic basis of human senescence, the field of allergic diseases in the elderly should be expanded to possibly find new diagnostic and therapeutic opportunities [25].

A large proportion of patients (30.4%) with allergies did not receive any treatment in this current study. Furthermore, a large percentage of participants did not have their allergies treated by a dermatologist or an ENT doctor, who are usually specialized in allergology. The most frequently consulted therapist was a GP (39.3%), which could be explained by the fact that there are more GPs than other specialists across the lower region of Bavaria. GPs are often the only available doctors for patients, especially in rural areas [26, 27].

Out of the 13 farmers who answered the question about allergies, 6 (44.4%) stated that they had an allergy. In contrast, it was previously observed that farmers suffered from less allergies and farming was recognized as a protective factor [14]. The specific role of modern agriculture for this relationship is not known. In another study, children living on very traditional farms had no hay fever or allergies. Children living on Hutterite farms, which use industrial farming practices, showed a high prevalence of asthma (> 15%) and allergic sensitization [28]. A possible explanation for why the protective factor of farming might no longer be present is because modern agriculture uses far more chemical substances, which also kill bacteria [23, 29]. Due to the small number of farmers participating in our study, we were unable to make any conclusions or comparisons to previous studies regarding the link between farming and allergies. However, we emphasize that additional studies are necessary to support this finding.

According to our results, there remains a large demand for diagnostics and therapies [30, 31] for allergies in rural Bavaria and many other parts of Europe [32]. An untreated insect or other allergies, such as certain food allergies (e.g., peanut), can lead to anaphylaxis and death [33–37]. In addition to allergic symptoms, a falsely treated case of hay fever can lead to the development of asthma, which will consequently lead to additional demand for therapy and increased healthcare costs [4].

In our study, the examined population lived in rural areas (i.e., countryside) of Bavaria, which would create the assumption that there are less allergies compared to the past in the United States [28]. Unexpectedly, the prevalence of allergies in this current study was higher than the prevalence of allergies found by the RKI in 2016 for Germany [4]. A major difference between our study and that conducted by the RKI was that the RKI had investigated the German population as a whole rather than focusing on the rural population. In total, 30.0% of the population of the RKI survey and 37.3% of our study group had at least one allergy. Furthermore, there were some differences in the findings regarding the prevalence of different allergies between the RKI survey and our study (contact dermatitis, 8.1% vs. 6.5%; insect venom allergy, 2.8% vs. 4.8%; food allergies, 4.7% vs. 5.2%). There are many possible explanations for these differences. The best explanation for the higher prevalence of allergies was the fact that only people who consulted doctors participated in this current study, whereas the population for the RKI health survey was randomly chosen [4]. Another very important difference is that our study sample was smaller, resulting in non-representative results. Furthermore, the higher prevalence might be explained by the lifestyle, age, and profession of the participants in rural Bavaria, which differs from other areas and cities across Germany.

Epidemiological studies regarding the health of an entire population of a country have not been performed frequently in the past because they are difficult to conduct. We were unable to identify any previous studies that were conducted to evaluate different allergy treatments in a general population; therefore, this study is the first to shed light on the way patients treat their allergies. Although the study population was not representative of the entire German population, we were able to find that the most frequently consulted doctor for the treatment of allergies was a GP (39.3%), and that a large number of allergies were not treated at all (30.4%). However, the high number of GP consultations in rural areas of Bavaria could also be due to the unavailability of other specialties in that area. Figures concerning the availabity of specialities in larger cities would most probably be different to the findings that were observed in this current study. In addition, this was one of the first studies conducted to provide a comprehensive overview about the prevalence and treatment of allergies in the Bavarian countryside. Medical care in rural areas have many challenges due to demographic changes and urbanization [26], and therefore, determining the need for treatment in rural areas is crucial.

There were several limitations for the interpretation of our study results. There was a selection bias because the study questionnaires were only distributed in doctors’ offices, and therefore, we only included participants who had already consulted a doctor. Therefore, a direct comparison between our results and that of the RKI survey, which had randomly selected participants, was not possible. Self-filled questionnaires may lead to social desirability and recall bias, which can also influence the results. In addition, the information for this study was obtained from only the participant responses and we did not conduct any blood or skin tests concerning their diagnosis or have access to information regarding their treatment from the doctor, which may have contributed to further biases in this study. However, in a study conducted by the RKI in the 1990s, questionnaires answered by patients can identify the prevalence of allergies more accurately than interviews conducted by treating doctors [38]. A reason for this is that patients only consult their doctor when they require treatment for their allergies. The presence of an allergy is not always communicated and can be determined more easily by a questionnaire.

An additional limitation was the duration of the study. The questionnaires were only distributed for 3 months (January–March), which might not be a sufficient period because pollen allergies are most common during this period anyway. The participants might therefore tend to emphasize answers for pollen allergies because this allergy was more common during the time of the questionnaire. The small number of participating farmers did limit the significance of our finding that farmers have a higher incidence of allergies than previously assumed. To support our results and findings, future studies should focus on the prevalence of allergies amongst farmers, be conducted in a wider population range (i.e., not only in doctors’ offices), and also address the treatment of allergies nationwide.

## Conclusion

The two main conclusions of our study were that the prevalence of allergies was high in rural areas and that most cases of allergies are not adequately treated. In order to reach more patients and increase their awareness regarding allergies and possible treatments, awareness and information campaigns may represent a possible solution. An improvement of patient-centered-care in allergology could lead to a reduction in the burden of allergic disease. As already used in other areas of dermatology, telemedicine might be a useful method for the treatment of allergies in rural areas in the future [38, 39].

## Abbreviations

CI: Confidence interval
ENT: Ear, nose, and throat specialist
GP: General practitioner
IgE: Immunoglobulin E
OR: Odds ratio
RKI: Robert Koch Institute
